# Epitweetr: Early warning of public health threats using Twitter data

**DOI:** 10.2807/1560-7917.ES.2022.27.39.2200177

**Published:** 2022-09-29

**Authors:** Laura Espinosa, Ariana Wijermans, Francisco Orchard, Michael Höhle, Thomas Czernichow, Pietro Coletti, Lisa Hermans, Christel Faes, Esther Kissling, Thomas Mollet

**Affiliations:** 1 European Centre for Disease Prevention and Control (ECDC), Stockholm, Sweden; 2 Epiconcept, Paris, France; 3 Stockholm University, Stockholm, Sweden; 4 Current affiliation: Aleia, Paris, France; 5 Hasselt University, Hasselt, Belgium; 6 Current affiliation: International Federation of Red Cross and Red Crescent Societies, Geneva, Switzerland

**Keywords:** early warning, Twitter, public health, machine learning, epidemic intelligence

## Abstract

**Background:**

The European Centre for Disease Prevention and Control (ECDC) systematically collates information from sources to rapidly detect early public health threats. The lack of a freely available, customisable and automated early warning tool using data from Twitter prompted the ECDC to develop epitweetr, which collects, geolocates and aggregates tweets generating signals and email alerts.

**Aim:**

This study aims to compare the performance of epitweetr to manually monitoring tweets for the purpose of early detecting public health threats.

**Methods:**

We calculated the general and specific positive predictive value (PPV) of signals generated by epitweetr between 19 October and 30 November 2020. Sensitivity, specificity, timeliness and accuracy and performance of tweet geolocation and signal detection algorithms obtained from epitweetr and the manual monitoring of 1,200 tweets were compared.

**Results:**

The epitweetr geolocation algorithm had an accuracy of 30.1% at national, and 25.9% at subnational levels. The signal detection algorithm had 3.0% general PPV and 74.6% specific PPV. Compared to manual monitoring, epitweetr had greater sensitivity (47.9% and 78.6%, respectively), and reduced PPV (97.9% and 74.6%, respectively). Median validation time difference between 16 common events detected by epitweetr and manual monitoring was -48.6 hours (IQR: −102.8 to −23.7).

**Conclusion:**

Epitweetr has shown sufficient performance as an early warning tool for public health threats using Twitter data. Since epitweetr is a free, open-source tool with configurable settings and a strong automated component, it is expected to increase in usability and usefulness to public health experts.

**Public health impact of this article**

**What did you want to address in this study?**

Twitter data can be used to detect outbreaks and public health threats. Epitweetr is a new open-source, tool for automated early detection of public health threats that uses Twitter data. We wished to compare the performance of epitweetr against the manual monitoring of Twitter for early detection of public health threats.

**What have we learnt from this study?**

Epitweetr has been shown to have an advantage over manually monitoring Twitter data in terms of being able to detect public health threats early. This type of tool has been shown to be useful to public health experts, and it is highly recommended that epitweetr be used to collect epidemic data in combination with existing tools.

**What are the implications of your findings for public health?**

Making epitweetr available publicly, and including several customisable settings allows users to adapt this tool to their specific needs and also further develop this tool. Since epitweetr has a strong automated component providing data in a timely manner, it can become a useful tool in the daily detection of infectious diseases, or other health threats, in public health settings.

## Introduction

The aim of the European Centre for Disease Prevention and Control (ECDC), a European Union (EU) agency, is to strengthen Europe’s defences against infectious diseases. Article 3 of the ECDC Founding Regulation, Decision Number 1082/2013/EU of the European Parliament and of the Council of 22 October 2013 on serious cross-border threats to health and the ECDC Strategy 2021–2027 have established the detection of public health threats as a core activity of ECDC.

The ECDC uses epidemic intelligence activities to collate information from a variety of sources, which is then validated and analysed. The aim is to rapidly detect and assess public health events, focusing on infectious diseases, to ensure the EU’s health security [1]. Currently, the ECDC monitors social media as part of its epidemic intelligence activities, in particular Twitter and Facebook due to their widespread use in some regions in the world and accessible text format [2]. In the past few years, around one third of signals detected by the ECDC through epidemic intelligence activities originated from social media [3]. These platforms are often updated by local, national, and international health authorities posting new information, which allows the ECDC to capture signals from small areas where media coverage is insufficient.

There have been previous attempts to use social media data for automated early detection of signals of public health threats [4-6], and a review of the use of Twitter for public health surveillance was published in December 2018 [7]. However, this review mainly targeted the monitoring of already detected outbreaks through Twitter, without fully covering monitoring of social media for early detection of public health threats. In addition, the authors stated that the geolocation of tweets through geotagging remained a major challenge. Several other studies have described the use of Twitter for outbreak investigation [8-10] or for understanding public perception of an epidemic [11,12], but these did not provide insights into the possible use of social media for automated event detection and real-time monitoring.

In the context of the coronavirus disease (COVID-19) pandemic, social media have become a key tool for sharing and disseminating data and information. In 2021, a scoping review examined studies related to COVID-19 and social media during the first year of the pandemic [13]. Surveillance and monitoring was one of the six themes extracted from these studies and according to the authors, no real-time surveillance monitoring had been developed for COVID-19 using social media data. Likewise, Lopreite and colleagues [14], retrospectively analysed Twitter data to uncover early warning signals of COVID-19 outbreaks in Europe in the winter season 2019/20. This showed the relevance and stressed the urgency of having these early warning systems in place to better identify public health threats that may proliferate almost undetected otherwise.

Noting the usefulness of having free, customisable and automated early warning tools using social media, the ECDC developed a prototype of an R-based tool in August 2019 for the early detection of public health threats using Twitter data. Twitter was selected from the different social media platforms due to both its widespread use in some regions of the world such as the European Union/European Economic Area (EU/EEA), and its free and easily accessible data application programming interface (API). The prototype focussed on a Public Health Event of International Concern (PHEIC) that received major attention in social media: the 2019 Ebola virus disease outbreak in the Democratic Republic of the Congo. The prototype was further extended in October 2019 and January 2020 by the inclusion of two other PHEICs: poliomyelitis and COVID-19. After the favourable results of this prototype, the ECDC developed a free, open-source tool named epitweetr to automatically monitor Twitter data for early warning of public health threats. Using a pre-determined but adaptable selection of keywords, this tool picks up new events of known public health threats, and using a broader set of keywords, it can also pick up new emerging health threats. The first version of this tool was published on the Comprehensive R Archive Network (CRAN) in October 2020 [15].

The main objective of our study is to evaluate epitweetr version 1 published in October 2020, a new automated, open-source, R-based tool for early detection of public health threats using Twitter data. The specific objectives are to assess the performance of the geolocation and signal detection algorithms used by epitweetr and to assess the performance of epitweetr in comparison with the manual monitoring of Twitter for early detection of public health threats.

## Methods

### Epitweetr

Epitweetr [15] collects Twitter data and metadata using the Twitter Standard Search API 1.1. It collects these data by sending queries according to a predetermined list of topics with related keywords. Throughout the time of this study period, 11 September to 30 November 2020, a list of 70 unique topics was used, focusing on notifiable or infectious diseases [16] (Supplement S1).

**Figure 1:**
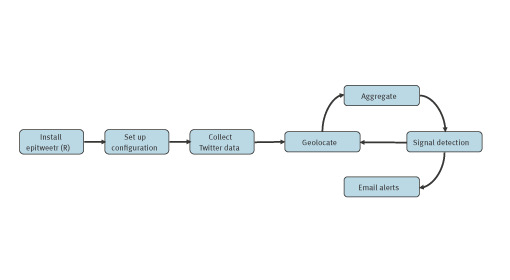
The epitweetr pipeline from installation to email alerts

In parallel with the Twitter data collection, epitweetr processes these data to geolocate tweets, aggregate them, detect signals and send these signals through email alerts (Figure 1) [17].

The geolocation process aims to collect tweet and user location, with the tweet location being the primary location used for the signal detection.

The geolocation process aims to collect tweet and user location, with the tweet location being the primary location used for the signal detection.

The tweet location is based on the location found in the tweet, retweet or quote text. In cases where there is no information in the retweet or quote, the retweeted or quoted text is used. Epitweetr extracts the tweet location in two steps. In the first step, epitweetr looks for words that could be location candidates. It transforms the tweet text into vectors (words) using fasttext [18], a well-known natural language processing technique [16]. Up to 157 languages can be chosen from fasttext; however, for this evaluation the four alphanumeric languages used in the EU/EEA with the highest number of tweets worldwide were selected (English, French, Portuguese and Spanish) due to the EU/EEA focus and to reduce the processing time [19]. Epitweetr uses a supervised machine learning algorithm which is automatically trained with labelled datasets. This algorithm detects parts of the text referring to geographic locations using locations from the GeoNames database [20]. The non-location words are obtained by extracting common words in fasttext models not included in the GeoNames database. In the second step, the text of these location candidates is matched against the GeoNames database using the Apache Lucene search engine [21], which implements a variant of vector space model (VSM) based on document-query similarity. In each of these steps, a score is allocated. The words with the higher score in the first step are selected as a candidate for the second step, and the highest matching score on the second step is chosen as tweet geolocation if it is higher than the threshold defined by the user. The higher the threshold score is, the lower the false positive rate is expected to be. Having a lower false positive rate, users should expect higher accuracy and specificity; however, a lower sensitivity is expected.

For the user location, the location metadata available from the API is extracted using the same process. The best user location will be selected, with user’s location at the time of the tweet a priority, followed by the self-declared user location or location as set in the public profile or the biography of the user.

**Figure 2:**
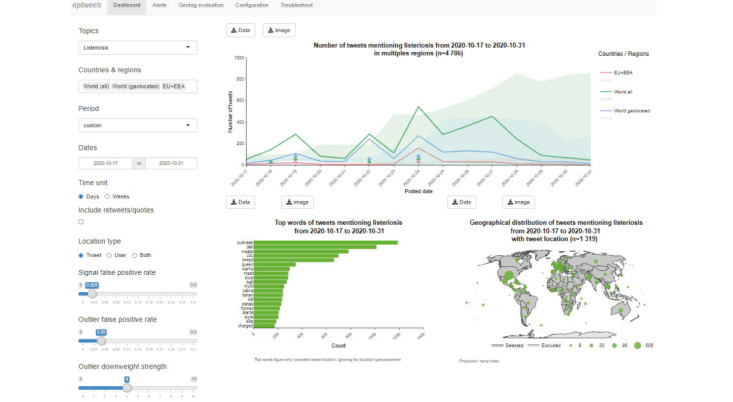
Epitweetr dashboard

The aggregation process creates the data shown in the three figures in the epitweetr dashboard (Figure 2) based on Shiny web application framework [22]: time series of tweets, map of tweet and/or user location, and the 20 most frequent words in the tweets.

Epitweetr detects signals (Supplement S2) [17], using the time series of previous tweets for the same topic and country. Epitweetr does not take into account retweets or quoted tweets for signal detection. The length of the time series used for calculating signals can be configured by the user, having a default value of 7 days which was also used in this evaluation. Each of the univariate time series is processed by a modified version of the Early Aberration Reporting System (EARS) algorithm [23], most commonly used for the detection of abnormalities over time, and as implemented in the R package ‘surveillance’ [24]. The algorithm calculates a threshold for the expected tweet count for each topic and location (national level or higher) as a given quantile of the predictive distribution. If the threshold is exceeded, a signal is created for that time series. Inspired by Farrington and colleagues [25], the estimation of the threshold of an aberration/signal also downweighs previous values if these are considered outliers.

These signals are sent out in email alerts with the following variables for each signal: date, hour, topic, region, top words, number of tweets, percentage of tweets from trusted Twitter users and configuration used to produce these signals (e.g., alert confidence or strength of previous signals’ downweight).

### Evaluating the geolocation algorithm

We randomly selected 1,200 of the tweets extracted by epitweetr and manually evaluated their primary geolocation to ascertain how accurate epitweetr was. Three hundred tweets per day were collected on 11, 16, 19 and 22 September 2020, generating a total of 1,200 tweets. These tweets were assigned to one of two experts in epidemic intelligence (LE and AW), who each evaluated 150 tweets per day. Each expert identified the best fitting geolocation at national and subnational level, if such information was available.

The accuracy of the geolocation algorithm in extracting the correct location and the performance of the geolocation algorithm in deciding which tweets contained extractable location information (hereafter referred as tweets with an extractable location) at national and subnational level were assessed by comparing the manual and epitweetr geolocations. For each tweet, we defined a positive hit when a location could be extracted from the tweet and a negative hit when no location could be extracted from the tweet. For positive hits, tweets were considered true positives or false positives depending on whether epitweetr assigned a location for these tweets or not. For negative hits, tweets were considered true negatives or false negatives depending on whether epitweetr did not or did assign a location for these tweets.

The following calculations were made regarding geolocation algorithm performance: accuracy, sensitivity, specificity, positive predictive value (PPV), negative predictive value (NPV) and prevalence (number of tweets from which location could be extracted) [26]. The accuracy for each tweet was calculated as 1 if the geolocation extracted by epitweetr matched the geolocation extracted manually and as 0 if these two geolocations did not match. The overall accuracy in per cent was calculated as the proportion of matches from the total number of tweets. We calculated accuracy at national and subnational level.


$$Accuracy\left(geolocation\right)\text{=}\frac{true\;positive\;geolocations}{all\;geolocations}\text{;}$$

$$Sensitivity\;(geolocation)\;\text{=}\frac{true\;positive\;geolocations}{\text{true positive geolocations + false negative geolocations}}\text{;}$$

$$Specificity\;(geolocation)\;\text{=}\frac{true\;negative\;geolocations}{\text{true negative geolocations + false positive geolocations}}\text{;}$$

$$PPV\;(geolocation)\;\text{=}\frac{true\;positive\;geolocations}{\text{true positive geolocations + false positive geolocations}}\text{;}$$

$$NPV\left(geolocation\right)\text{=}\frac{true\;negative\;geolocations}{\text{true negative geolocations + false positive geolocations}}\text{;}$$

$$Prevalence\;(geolocation)\;\text{=}\frac{true\;positive\;geolocations\;+\;false\;negative\;geolocations}{\text{all geolocations}}\text{;}$$

The average of the results from both experts manually assessing tweets for geolocation was calculated. Additionally, these calculations were made according to the geolocation score dividing tweets in two groups: tweets with tweet geolocation score below 10, and tweets with tweet geolocation score above 10 (the default minimum score for accepting geolocations predicted by epitweetr).

Furthermore, we assessed the most frequent errors made by the algorithm in extracting the correct location and grouped them in two categories: tweets mentioning the president of the United States (US) (tweets mentioning the US president were not geolocated in the US as expected, since the current geolocation algorithm does not take into account persons as keywords for location) and/or a ‘well established location’ that was not found by epitweetr (e.g. country names, country populations, US state names and capital cities). The same previously mentioned calculations were made to evaluate what would have been the performance of epitweetr geolocation algorithm if these locations had been extracted correctly by epitweetr.

### Evaluating the signal detection algorithm

We assessed alerts (number of tweets that exceed the threshold) generated by epitweetr during working days between 19 October and 30 November 2020 to determine which were validated events. We defined a signal as an alert in which top words and other information included in the email suggested it fulfilled ECDC epidemic intelligence selection criteria based on International Health Regulations and Decision no. 1082/2013/EU. An event was an epitweetr signal that was validated (i.e. deemed accurate and reliable information, and confirmed by or originated from an official source).

Following the epidemic intelligence steps [27], we evaluated only the signals. We investigated the events or group of tweets that could have triggered those signals and validated the information. On occasion, after retrieving the events, the signals were discarded due to additional information provided in the tweets or the triggering events which did not fulfil ECDC epidemic intelligence selection criteria.

We defined the general PPV (PPV_g_) and specific PPV (PPV_s_) as:


$$Prevalence\;(geolocation)\;\text{=}\frac{true\;positive\;geolocations\;+\;false\;negative\;geolocations}{\text{all geolocations}}\text{;}$$

$${\text{PPV}}_{\text{g}}\text{=}\frac{\text{events/true signals}}{\text{events/true signals+false signals+not evaluated alerts}}\text{;}$$

where *events* were epitweetr alerts that fulfilled ECDC epidemic intelligence selection criteria (i.e. signals) and were validated, *false signals* were evaluated alerts that seemed to fulfil ECDC epidemic intelligence selection but were discarded after evaluating the triggering event(s) as these did not fulfil the ECDC epidemic intelligence selection criteria and/or were not validated, and *not evaluated alerts* were alerts detected by epitweetr algorithm that did not fulfil ECDC epidemic intelligence selection criteria based on the most frequent words, topic, location and number of tweets for which reason these were not evaluated.

To summarise, epitweetr emails contained alerts (i.e. an unexpected increase in the number of tweets for a specific topic, place and time). Some of these alerts were not evaluated and some were evaluated manually by epidemic intelligence experts. From the evaluated alerts, those fulfilling ECDC epidemic intelligence selection criteria were considered true signals or events, and those discarded after the manual evaluation (not validated and/or not fulfilling ECDC epidemic intelligence selection criteria after further assessment) were considered false signals.

The PPV_g_ considered all alerts detected by epitweetr whereas PPV_s_ considered only signals further assessed by the experts.

### Evaluating epitweetr

We developed a study protocol to evaluate the sensitivity, specificity and timeliness of epitweetr in comparison to the manual monitoring of Twitter for early detection of public health threats (Supplement S3).

It is difficult to evaluate the classification accuracy of the generated events using the two methods, because no independent gold standard exists and there is no available information on all events that should be detected by both methods. We used instead an inter-rater agreement (IRA) measure between the two methods as a relative definition of sensitivity.

Since the estimation of the specificity was not feasible in this context, we calculated the PPV as the proportion of signals from each method corresponding to a validated event.

From the total number of events detected by both methods, we manually deduplicated these according to the topic provided by epitweetr and subtopic manually extracted by the two experts to eliminate events captured on different days and/or locations (e.g. hantavirus case geolocated by epitweetr in Argentina, South America and America that corresponded to the same event), providing the number of unique events.

We defined the timeliness as the difference between the validation time of unique events found by epitweetr and manual monitoring of Twitter. We performed a descriptive analysis, including measures of central tendency and variability. Likewise, we performed a significance test using the Wilcoxon signed rank test where the null hypothesis assumed there was no true difference and the alternative hypothesis assumed epitweetr had earlier validation times than the manual method. Level of significance was set to p < 0.05.

## Results

### Geolocation algorithm

At national level, the epitweetr geolocation algorithm had an overall accuracy of 30.1%, while at subnational level this was 25.9% (Table 1). From the 1,200 tweets, 774 tweets were considered by epitweetr to have an extractable location. Of these, the geolocation score ranged from 1.8 to 29.8 with a median of 9.9 and interquartile range (IQR: 11.5 – 8.2).

**Table 1:** Tweet location indicators determined from the results of the epitweetr geolocation algorithm, 11–22 September 2020 (n = 1,200)

Level		All results		Excluding tweets with scores lower than 10	
		National	Subnational	National	Subnational
**Total number of tweets**		**(n = 1,200)**	**(n = 1,200)**	**(n = 804)**	**(n = 804)**
Extracting correct location	Correct hits	361	311	292	283
	Accuracy	30.1%	25.9%	36.3%	35.2%
Detecting tweets with extractable location	Sensitivity	72.6%	72.2%	56.6%	56.2%
	Specificity	51.6%	50.6%	69.2%	68.3%
	PPV	74.9%	74.2%	75.7%	74.9%
	NPV	48.6%	48.1%	48.6%	48.1%
	Prevalence	66.6%	66.3%	62.8%	62.7%

NPV: negative predictive value; PPV: positive predictive value.

After correcting the geolocation errors linked to the mention of the US president (n = 281 tweets), we observed 38.5% accuracy, 76.0% sensitivity, 51.6% specificity and 75.8% PPV at national level. Likewise, after correcting the geolocation errors linked to ‘well established locations’ (n = 371), which also included country names, country populations, and capitals, we observed 52.4% accuracy, 88.8% sensitivity, 51.5% specificity and 78.5% PPV.

### Signal detection algorithm

During the study period from 19 October to 30 November 2020, 11,313 alerts were detected by epitweetr from which 448 were signals. From these evaluated signals, 334 were events and 114 were false signals.

From these 448 evaluated signals, 63 were related to COVID-19, including 48 events and 15 false signals. In addition, 49 of the 448 signals had only one tweet, including 24 events and 25 false signals.

Overall, the PPV_g_ was 3.0% and the PPV_s_ was 74.6%. The PPV_s_ for COVID-19 related events and for other events were 76.2% and 74.3%, respectively.

### Evaluating epitweetr

Data were collected from 19 October to 30 November 2020, to reach the minimum sample size. Overall, 570 signals were evaluated, including 122 signals detected by the manual method, 297 signals detected by epitweetr and 151 signals detected by both methods. From these, 157 were related to COVID-19, including 120 signals detected by the manual method, 24 signals detected by epitweetr and 39 signals detected by both methods.

Overall, 454 events fulfilling the ECDC epidemic intelligence selection criteria were detected, including 120 events detected by the manual method, 185 events detected by epitweetr and 149 events detected by both methods. From these, 157 were related to COVID-19, including 94 events detected by the manual method, 24 events detected by epitweetr and 39 events detected by both methods.

The number of signals and events, IRA and PPV of both methods are presented in Table 2.

**Table 2:** Number of signals and events from epitweetr and manual monitoring of tweets, 19 October–30 November 2020 (n = 570)

Variables	Manual monitoring	epitweetr
Number of signals	273	448
Number of events	269	334
IRA (95% CI)	47.9% (43.8­–52.0)	78.6% (75.2–82.0)
PPV (95% CI)	97.9% (95.8–99.9)	74.6% (70.5–78.6)

IRA: inter-rater agreement; PPV: positive predictive value.

**Figure 3:**
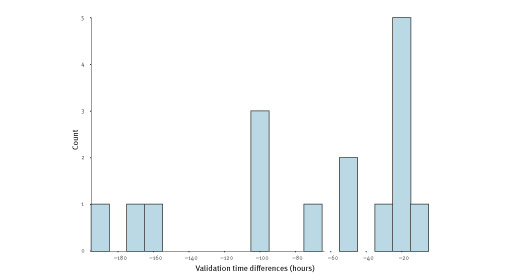
Differences in the validation time of common events between epitweetr and manual monitoring of tweets, 19 October–30 November 2020

A total of 16 unique events were found by both methods, including 10 events related to COVID-19. The median of the validation time differences was -48.6 hours (IQR: −102.8 to −23.7), showing a faster validation of common events by epitweetr. Figure 3 shows the distribution of the validation time differences in hours.

The Wilcoxon signed rank test showed that the validation time difference is significantly smaller than zero (p<0.05), meaning that validated events were detected by epitweetr earlier than those detected using manual monitoring.

## Discussion

In this article we present the evaluation of epitweetr, a new automatised, open-source, R-based tool for early detection of public health threats using Twitter data. This tool was developed after finding a lack of such a tool and performing a feasibility study through a non-automatised prototype.

Previous studies have shown the importance of having an appropriate geolocation when using social media data to better understand where the event or threat is happening. We focused our geolocation evaluation on the tweet geolocation since it is more relevant to provide accurate information related to the event rather than to the user. The tweet geolocation evaluation showed an approximate 30% accuracy at national level, which was ca. 4% higher than at subnational level. The majority of the wrongly geolocated tweets were related to a few recurrent errors from the algorithm such as US president and population citizens not being recognised (e.g. Trump, American, Chilean), common words getting high priority (e.g. ‘real’ for the location Ciudad Real, Spain) or well established locations not being recognised (e.g. Venezuela). Adding a supervised learning layer to the existing algorithm, the user could easily improve this by training the algorithm and thus increasing substantially epitweetr’s accuracy and specificity. This was seen in the increased accuracy at national level from 30.1% to 52.4% when a group of ‘well established locations’ could be correctly identified. This naturally also increased the sensitivity (up to 88.8%). However, the PPV only increased slightly (from 74.9% to 75.8% and 78.5% respectively) as some of these tweets had already been assigned a location even if it was the wrong location.

Epitweetr users can modify the threshold used by the geolocation algorithm to prioritise sensitivity or accuracy and specificity. Our evaluation proved that using a score above 10 as threshold increased the accuracy from 30.1 to 36.3% and the specificity from 51.6% to 69.2%, but it also decreased the sensitivity as expected.

We decided to use the Early Aberration Reporting System (EARS) as a baseline for the signal detection algorithm since it is a well-established methodology [28]. The initial evaluation of the modified EARS algorithm stressed the importance of the initial selection criteria and relevance of the further assessment of epitweetr alerts and signals shown by the huge difference between PPV_g_ (referring to all alerts) and PPV_s_ (referring only to the alerts manually assessed). Likewise, having an adaptable system to adjust other parameters such as false positive rate and sensitivity improves the results. Epitweetr is adaptable due to it having configurable settings that can be adjusted by topic

This modified EARS algorithm used by epitweetr for signal detection had a much higher sensitivity in comparison with the manual monitoring method and allowed small signals containing only one tweet to be detected. In general, epitweetr detected more events than the manual monitoring method, and did this in a timelier manner. When comparing the performance of epitweetr and manual monitoring for the COVID-19 signals and events, however, the latter detected more events. This can be explained by the fact that COVID-19 became a much-tweeted topic and the query used was too generic to detect these specific events. Creating more specific queries for COVID-19 would have allowed epitweetr to detect more events. This is relevant when a new event or threat is being monitored since a generic query can be used in the early stages and more specific queries should be developed once the event becomes more popular.

Epitweetr showed a lower positive predictive value in comparison with manual monitoring, as was expected for this signal detection algorithm in which sensitivity was prioritised over specificity. Epitweetr was developed to detect small signals so having a very sensitive tool was a priority. There are configurable settings that allow epitweetr users to modify the false positive rate of the tool. Furthermore, the positive predictive value of epitweetr could be increased by combining supervised and unsupervised learning to continuously train the model and adapt to possible concept drifts in Twitter streams [29] without jeopardising the sensitivity and IRA achieved by the underlying signal detection algorithm.

We believe that having configurable settings increases the flexibility of the tool and its ability to adapt to different uses. The dashboard of epitweetr is intended for testing these settings before epitweetr users decide which values to use in their context. This decision will depend on the resources available, which relates to the specificity of the tool (e.g. experts available to assess all signals detected by epitweetr, including possible false signals) and the granularity required, which relates to the sensitivity of the tool (e.g. which would be the consequences of missing a small signal).

Epitweetr has been implemented by the ECDC as an additional source of information for detecting in a timely and automated manner public health threats. In addition, the Italian National Institute of Health and the World Health Organization Eastern Mediterranean Office are using or testing epitweetr, and other institutions are investigating how to integrate epitweetr in the existing tools and processes [30]. Since October 2020, the ECDC has integrated epitweetr into its epidemic intelligence activities and standard operation procedures by making it one of the several sources used for screening, the first step of the epidemic intelligence process. Epitweetr is now used for the routine screening of public health threats within the ECDC mandate, specific screening of events, such as the COVID-19 pandemic, with generic queries and subqueries on specific topics (e.g. outbreaks or vaccines). It is also used for monitoring mass gathering events, setting up keywords for syndromes, symptoms and diseases from one week before the start to one week after the end of the event.

In other epidemic intelligence tools, screening of social media sources is not broadly integrated or includes Twitter messages from a predefined list of users, collecting only a limited amount of information. Epitweetr provides a holistic approach of collecting data from Twitter by collecting and processing all tweets, retweets and quoted tweets publicly available according to the keywords indicated by the user. By default, epitweetr only considers tweets for the signal detection, but it can be easily configured to consider also retweets and quoted tweets. Epitweetr should thus be considered to be one of the many resources supporting the early phases of epidemic intelligence activities.

The main limitation of epitweetr relates to the variation in Twitter data dynamics (e.g. new keywords needed if concepts/words used by Twitter users change) and different scopes within early detection of threats that epitweetr users may have. This has been overcome by adding most of the parameters as configurable settings that can be changed not only for the tool itself but also depending on the topic. Likewise, the dashboard facilitates this decision by showing the immediate results of choosing different parameters. Nonetheless, epitweetr must be used in combination with other epidemic intelligence tools since its sole use would have limited benefit for epidemic intelligence activities compared with using it in combination with other sources and tools. Epitweetr was not developed as a stand-alone tool for epidemic intelligence but as one of its many resources.

The results of this evaluation have shown some areas for improvement: automated signal analysis and categorisation, based on annotated signals by the user; improvement of the performance of tweet text location, based on annotated tweet text locations by the user; real-time performance matrix of the geolocation algorithm and management of signals with only one tweet, among others. In 2021, a new version of epitweetr was developed addressing these areas. In addition, this new version of epitweetr has a more efficient and easily searchable data architecture.

## Conclusion

Epitweetr has been shown to perform sufficiently well for early detection of public health threats using Twitter data. This type of tool has proven to be useful to public health experts and it is highly recommended it be used in epidemic intelligence activities in combination with other existing tools for an improved synergy. Moreover, making epitweetr available via a public repository with several customisable settings allows other users to adapt the tool to their specific needs and, even, further develop this tool. Additionally, since epitweetr has a strong automated component providing outputs in a timely manner, we believe it can become a useful tool in the daily public health practice of infectious disease event and threat detection.

## Supplementary Data

287000 Supplementary Material
