# Erratum for Euro Surveill. 2022;27(39)

**DOI:** 10.2807/1560-7917.ES.2022.27.44.221103e2

**Published:** 2022-11-03

**Authors:** Eurosurveillance editorial team

**Affiliations:** 1 European Centre for Disease Prevention and Control (ECDC), Stockholm, Sweden

In the article ‘Epitweetr: Early warning of public health threats using Twitter data’ by Espinosa et al. published on 29 September 2022, the formula for general PPV was wrong in the published article because of a technical error The formula was corrected and replaced on 28 October 2022, and we apologise for any inconvenience this error may have caused.

