# Supplementary material for: Epitweetr: Early warning of public health threats using Twitter data
Source: Euro Surveill. 2022 Sep 29;27(39):2200177. doi: 10.2807/1560-7917.ES.2022.27.39.2200177 (PMC9524055; doi:10.2807/1560-7917.ES.2022.27.39.2200177)
Supplement: Supplementary Material [file 2200177_ESPINOSA_Supplementary_material.pdf]

This supplementary material is hosted by *Eurosurveillance* as supporting information alongside the article ‘Epitweetr: Early warning of public health threats using Twitter data’, on behalf of the authors, who remain responsible for the accuracy and appropriateness of the content. The same standards for ethics, copyright, attributions and permissions as for the article apply. Supplements are not edited by *Eurosurveillance* and the journal is not responsible for the maintenance of any links or email addresses provided therein.

Supplementary material

Suplement S1. List of 70 unique topics

The 70 unique topics used for this study, by alphabetical order, were:

1. Anthrax
2. Antimicrobial resistance
3. Avian influenza
4. Bioterrorism
5. Botulism
6. Brucellosis
7. Campylobacteriosis
8. Chickenpox
9. Chikungunya
10. Chlamydiosis
11. Cholera
12. Clostridium difficile
13. Coronavirus disease (COVID-19)
14. Creutzfeldt-Jakob disease
15. Crimean-Congo haemorrhagic fever
16. Cryptosporidiosis
17. Dengue
18. Diphtheria
19. Ebola
20. Echinococcosis
21. Foodborne diseases
22. Giardiasis
23. Gonorrhoea
24. Haemophilus influenza
25. Haemorrhagic fever
26. Hantavirus
27. Healthcare-associated infections
28. Hepatitis A
29. Hepatitis B
30. Hepatitis C
31. HIV/AIDS
32. Infectious diseases
33. Lassa fever
34. Legionnaires’ disease
35. Leptospirosis
36. Listeriosis
37. Lyme disease
38. Lymphogranuloma venereus
39. Malaria
40. Measles
41. Meningococcal disease

42. Middle East Respiratory Syndrome (MERS-CoV)
43. Mumps
44. Pertussis
45. Plague
46. Pneumococcal disease
47. Poliomyelitis
48. Q fever
49. Rabies
50. Rift Valley fever
51. Rubella
52. Salmonellosis
53. Severe Acute Respiratory Syndrome (SARS)
54. Seasonal influenza
55. Shigellosis
56. Smallpox
57. Syphilis
58. Tetanus
59. Tick-borne encephalitis
60. Toxoplasmosis
61. Trichinellosis
62. Tuberculosis
63. Tularaemia
64. Typhoid fever
65. Waterborne diseases
66. West Nile virus
67. Yellow fever
68. Yersiniosis
69. Zika
70. Zoonoses

Supplement S2. Details of epitweetr signal detection algorithm

The algorithm is applied on the counts from the past seven 24-hour blocks prior to the current 24-hour block of the signal detection. The running mean and the running standard deviation are calculated:

$$\bar{y}_0 = \frac{1}{7}\sum_{t=-7}^{-1} y_t \quad and \quad s_0^2 = \frac{1}{7-1}\sum_{t=-7}^{-1}(y_t - \bar{y}_0)^2,$$

where y_t,t = …, −2, −1, 0 denotes the observed count data time series with time index 0 denoting the current block. Furthermore, the time index −7, …, −1 denote the seven blocks prior to the current block.

Under the null hypothesis of no spikes, it is assumed that the y_t are identically and independently $N(\mu,\sigma^2)$ distributed with unknown mean μ and unknown variance σ^2. Hence, the upper limit of a simple one-sided (1− α)× 100% plug-in prediction interval for y_0 based on y−7, …, y−1 is given as

$$U_o = \bar{y}_0 + z_{1-\alpha} \times s_0,$$

where $z_{1-\alpha}$ is the (1 − α)- quantile of the standard normal distribution. An alert is raised if $y_0 > U_0$. Using α=0.025, it corresponds to investigating if y_0 exceeds the estimate for the mean plus 1.96 times the standard deviation. However, as pointed out by Allévius and Höhle (2017), the correct approach would be to compare the observation to the upper limit of a two-sided 95% prediction interval for y_0, because this considers both the sampling variation of a new observation and the uncertainty originating from the parameter estimation of the mean and variance. Hence, the statistical appropriate form is to compute the upper limit by

$$U_0 = \overline{y_0} + t_{1-\propto}(7-1) \times s_0 \times \sqrt{1+\frac{1}{7}},$$

where $t_{1-\alpha}(k-1)$ denotes the 1 − α quantile of the t-distribution with k − 1 degrees of freedom.

If previous signals are included without modification in the historic values when calculating the running mean and standard deviation for the signal detection, then the estimated mean and standard deviation might become too large. This may mean that important current signals will not be detected. To address this issue, epitweetr downweights previous signals, such that the mean and standard deviation estimation is adjusted for such outliers using an approach similar to that used in the Farrington et al. (1996). Historic values that are not identified as previous signals are given a weight of "1". Similarly, historic values identified as signals are given a weight lower than one and a new fit is performed using these weights. Details on the downweighting procedure can be found in Annex I of this user documentation.

Signal detection is carried out based on "days", which are moving windows of 24 hours, moving according to the detect span. The baseline is calculated on these "days" from -1 to -7, considering the current "day" as zero.

A key attribute of signal detection is the ability of an algorithm to detect true threats or events without overloading the investigators with too many false positives. In this way, the alpha parameter determines the threshold of the detection interval. If the alpha is high, then more potential signals are generated and if the alpha is low fewer potential signals are generated (but potential threats or events could be missed). The setting of the alpha is often done empirically and depends also on the resources of those investigating the signals and the importance of missing a potential threat or event. Currently, this attribute is merely statistically based only considering previous parameters explained in the algorithm.

To account for multiple testing, a Bonferroni correction for multiple testing can be applied. For country-specific signal detection, as a default, the alpha is divided by the number of countries. For continent-specific signal detection, the alpha is divided by the number continents.

Supplement S3. Additional methods: epitweetr evaluation

The manual monitoring (hereafter referred to as the manual method) consisted of screening twice a day most recent tweets posted by a list of over 100 validated Twitter users followed by ECDC EI team. In this method, we defined a signal as a tweet that fulfils ECDC criteria and required further action (e.g., validation of the information). The time of this tweet was recorded as the signal time and, in case several Twitter accounts were tweeting about the same topic in the same screening round, the earliest set the signal time.

The epitweetr screening consisted of screening twice a day email alerts (i.e., unexpected increase in the number of tweets by topic, location and time) sent by epitweetr at approximately 4.30 Central European Time (CET) and 13.30 CET. In this method, we defined a signal as an alert for a specific topic, location and time which top words and other information included in the email suggested it fulfilled ECDC criteria. The time of the earliest alert of the day was recorded as the signal time.

In both epitweetr and the manual method, an event was a validated signal deemed trustful and reliable by an official source. The approximate time at which this validation was done was recorded as the event time, both in case of positive and negative validation.

Two EI experts screened during alternate weeks Twitter data twice a day using both methods and recorded signals and events detected by each method until the minimum sample size was achieved.

Considering there is no prior estimate available for the sensitivity and specificity and having a maximal marginal error of 0.15 for sensitivity and specificity, we defined the minimum sample size as:

$$n \geq \frac{z^2}{4d^2};$$

where *n* is the minimum sample size, *z* is the 97·5% percentile of the standard normal distribution, and *d* is the maximal marginal error.

In order to achieve a minimum sample size, a time period with the following criteria was selected: at least 43 different events found by any of the methods, at least 43 signals found by each method and at least 10 events found by both methods.

Since it is difficult to evaluate the classification accuracy of the generated events by the two methods, because no independent gold standard exists and there is no available information on all events that should be detected by both methods, we used instead an inter-rater agreement (IRA) between the two methods as a relative definition of sensitivity[1]. We defined the IRA of the manual method (IRA_m) and the IRA of epitweetr (IRA_e), with their 95% confidence interval (CI), as:

$$IRA_m = \frac{a+c}{a+b+c} \qquad CI: \left[IRA_m \pm 1.96\sqrt{\frac{IRA_o(1-IRA_m)}{a+b+c}} \right];$$

$$IRA_e = \frac{a+b}{a+b+c} \qquad CI: \left[IRA_e \pm 1.96\sqrt{\frac{IRA_e(1-IRA_e)}{a+b+c}} \right];$$

where *a* was the number of events detected by both methods, *b* was the number of events detected only by epitweetr, and *c* the number of events detected only by the manual method.

Since the estimation of the specificity was not feasible in this context, we calculated the PPV as the proportion of signals corresponding to a validated event. We defined the manual method PPV (PPV_m) and the epitweetr PPV (PPV_e), with their 95% CI, as:

$$PPV_m = \frac{N_m^{ev}}{N_m^{sig}} \qquad CI: \left[PPV_m \pm 1.96\sqrt{\frac{PPV_m(1-PPV_m)}{N_m^{sig}}} \right];$$

$$PPV_e = \frac{N_e^{ev}}{N_e^{sig}} \qquad CI: \left[PPV_e \pm 1.96\sqrt{\frac{PPV_e(1-PPV_e)}{N_e^{sig}}} \right];$$

where N_m^{ev} was the number of events detected by the manual method, N_m^{sig} the number of signals detected by the manual method, N_e^{ev} the number of events detected by epitweetr, and N_e^{sig} the number of signals detected by epitweetr.

We defined the timeliness as the difference between the validation time of events found by epitweetr and manual method. In case an event was detected in several days, only the earliest was kept for the analysis. We performed a descriptive analysis, including measures of central tendency and variability. Likewise, we performed a significance test using the signed rank test where the null hypothesis assumed there was no true difference and the alternative hypothesis assumed epitweetr had earlier validation times than the manual method.

References

1. Fleiss L. Statistical methods for rates and proportions. New York: Wiley; 1981. p. 212-36.
